# Supplementary material for: The perils and promises of fact-checking with large language models
Source: Front Artif Intell. 2024 Feb 7;7:1341697. doi: 10.3389/frai.2024.1341697 (PMC10879553; doi:10.3389/frai.2024.1341697)
Supplement: Supplementary file 1 [file Data_Sheet_1.PDF]

Supplementary Material for The Perils & Promises of Fact-checking with Large Language Models

Dorian Quelle* **and Alexandre Bovet**

Correspondence*:
Dorian Quelle
dorian.quelle@uzh.ch

1 SUPPLEMENTARY TEXT

We present below the full details of the four examples shown in Figs. 2 & 3 of the main text. We include the full input (prompt), all the Google search queries performed by the agent, and all the previews of the retrieved Google results that the agent used. We also show the final output of the agent and the corresponding Politifact ruling.

1.1 Example 1 - Ted Cruz is mathematically out of winning the race.

Input: You're a fact-checking expert. Evaluate the truthfulness of this statement: '"Ted Cruz is mathematically out of winning the race."' made by Donald Trump on 4/20/2016. Use Google for research. Consider your sources, context, and date while assessing. To answer, return 'Final Answer: verdict, reason. You must respond with a valid verdict ('pants-fire','false', 'mostly-false', 'half-true', 'mostly-true', or 'true') or 'uncertain', providing reasoning and citing sources by providing the domain of pertinent search results.

Google Search Query: Ted Cruz Donald Trump delegate count April 20 2016

2016 Delegate Count and Primary Results - The New York Times - According to the Associated Press, Donald J. Trump and Hillary Clinton have each won enough ... Donald J. Trump. Earned. Unpledged. 1,447. 95. Ted Cruz. `https://www.nytimes.com/interactive/2016/us/elections/primary-calendar-and-results.html`

The Real Magic Number That Donald Trump Needs to Hit Is Likely ... - Experts weigh in on the delegate count needed for the GOP nomination. ByMEGHAN KENEALLY. April 20, 2016, 8:26 AM. 4:02 ... Ted Cruz or Gov. `https://abcnews.go.com/Politics/real-magic-number-donald-trump-hit-lower-1237/story?id=38539256`

2016 Republican Party presidential primaries - Wikipedia - Cruz won four Western contests and won in Wisconsin, keeping open a credible path to denying Trump the nomination on first ballot with 1,237 delegates. However, ... `https://en.wikipedia.org/wiki/2016_Republican_Party_presidential_primaries`

Ted Cruz Has Bad New York Primary Night - Ted Cruz won less than 15% of the vote and no delegates at the ... It was not a good night for Ted Cruz ... Pamela Engel. Apr 20, 2016, 7:02 AM PDT. A ... `https://www.businessinsider.com/ted-cruz-new-york-primary-results-trump-2016-4`

Ted Cruz 2016 presidential campaign - Wikipedia - In late April, while trailing Trump heavily in the delegate count, Cruz announced that his vice presidential running mate would be former-candidate Carly ... https://en.wikipedia.org/wiki/Ted_Cruz_2016_presidential_campaign

2016 Republican Nomination Calendar Released for Primaries | Time - Oct 2, 2015 ... Ted Cruz, real estate magnate Donald Trump, former Florida Gov. ... See also: Up-to-date delegate counts each Republican candidate. https://time.com/4059030/republican-primary-calendar-2016-nomination-convention/

GOP Can Prepare for Open Convention by Studying 1976 - Roll Call - Posted April 20, 2016 at 10:00am ... but a Trump surrogate has already accused the Ted Cruz campaign of “bribing” delegates — something that if true, ... https://www.rollcall.com/2016/04/20/gop-can-prepare-for-open-convention-by-studying-1976/

Ted Cruz needs a contested convention to win — and here’s how he ... - Updated 7:24 PM EDT, Wed April 20, 2016. Link Copied! Ad Feedback. Video Ad Feedback. Ted Cruz says Donald Trump won’t get 1,237 delegates. https://www.cnn.com/2016/04/20/politics/ted-cruzs-northeastern-survival-plan/index.html

What we learned from the New York primary | US elections 2016 ... - Apr 20, 2016 ... With nearly 95% of the vote counted, Trump leads with 60.1% of the vote, with Ohio governor John Kasich at 25.2% and Texas senator Ted Cruz ... https://www.theguardian.com/us-news/2016/apr/20/hillary-clinton-donald-trump-new-york-primary-what-we-learned

Trump blasts ’rigged’ rules on picking Republican delegates | Reuters - April 11, 2016 6:03 AM Updated 7 years ago ... Trump has 743 bound delegates to 545 for Cruz, according to an Associated Press count, in the battle for the ... https://www.reuters.com/article/us-usa-election/trump-blasts-rigged-rules-on-picking-republican-delegates-idUSKCN0X81HE

Google Search Query: Ted Cruz Donald Trump delegate count April 20 2016

2016 Delegate Count and Primary Results - The New York Times - According to the Associated Press, Donald J. Trump and Hillary Clinton have each won enough ... Donald J. Trump. Earned. Unpledged. 1,447. 95. Ted Cruz. https://www.nytimes.com/interactive/2016/us/elections/primary-calendar-and-results.html

The Real Magic Number That Donald Trump Needs to Hit Is Likely ... - Experts weigh in on the delegate count needed for the GOP nomination. ByMEGHAN KENEALLY. April 20, 2016, 8:26 AM. 4:02 ... Ted Cruz or Gov. https://abcnews.go.com/Politics/real-magic-number-donald-trump-hit-lower-1237/story?id=38539256

2016 Republican Party presidential primaries - Wikipedia - Cruz won four Western contests and won in Wisconsin, keeping open a credible path to denying Trump the nomination on first ballot with 1,237 delegates. However, ... https://en.wikipedia.org/wiki/2016_Republican_Party_presidential_primaries

Ted Cruz Has Bad New York Primary Night - Ted Cruz won less than 15% of the vote and no delegates at the ... It was not a good night for Ted Cruz ... Pamela Engel. Apr 20, 2016, 7:02 AM PDT. A ... https://www.businessinsider.com/ted-cruz-new-york-primary-results-trump-2016-4

Ted Cruz 2016 presidential campaign - Wikipedia - In late April, while trailing Trump heavily in the delegate count, Cruz announced that his vice presidential running mate would be former-candidate Carly ... `https://en.wikipedia.org/wiki/Ted_Cruz_2016_presidential_campaign`

2016 Republican Nomination Calendar Released for Primaries | Time - Oct 2, 2015 ... Ted Cruz, real estate magnate Donald Trump, former Florida Gov. ... See also: Up-to-date delegate counts each Republican candidate. `https://time.com/4059030/republican-primary-calendar-2016-nomination-convention/`

GOP Can Prepare for Open Convention by Studying 1976 - Roll Call - Posted April 20, 2016 at 10:00am ... but a Trump surrogate has already accused the Ted Cruz campaign of "bribing" delegates — something that if true, ... `https://www.rollcall.com/2016/04/20/gop-can-prepare-for-open-convention-by-studying-1976/`

Ted Cruz needs a contested convention to win — and here's how he ... - Updated 7:24 PM EDT, Wed April 20, 2016. Link Copied! Ad Feedback. Video Ad Feedback. Ted Cruz says Donald Trump won't get 1,237 delegates. `https://www.cnn.com/2016/04/20/politics/ted-cruzs-northeastern-survival-plan/index.html`

What we learned from the New York primary | US elections 2016 ... - Apr 20, 2016 ... With nearly 95% of the vote counted, Trump leads with 60.1% of the vote, with Ohio governor John Kasich at 25.2% and Texas senator Ted Cruz ... `https://www.theguardian.com/us-news/2016/apr/20/hillary-clinton-donald-trump-new-york-primary-what-we-learned`

Trump blasts 'rigged' rules on picking Republican delegates | Reuters - April 11, 2016 6:03 AM Updated 7 years ago ... Trump has 743 bound delegates to 545 for Cruz, according to an Associated Press count, in the battle for the ... `https://www.reuters.com/article/us-usa-election/trump-blasts-rigged-rules-on-picking-republican-delegates-idUSKCN0X81HE`

Google Search Query: Republican primary delegate count by candidate April 20 2016

2016 Delegate Count and Primary Results - The New York Times - Delegate totals include unpledged delegates, also known as superdelegates, who are free to support any candidate at the party conventions. `https://www.nytimes.com/interactive/2016/us/elections/primary-calendar-and-results.html`

2016 Republican Party presidential primaries - Wikipedia - However, Trump scored landslide victories in New York and five northeastern states in April, before taking every delegate in the Indiana primary on May 3. `https://en.wikipedia.org/wiki/2016_Republican_Party_presidential_primaries`

Election Results | Washtenaw County, MI - Official Election Results; Official List of Candidates - June 5, ... August 4, 2020 State Primary ... Precinct Delegate Allocation - Republican Party ... `https://www.washtenaw.org/314/Election-Results`

2016 Republican Nomination Calendar Released for Primaries | Time - Oct 2, 2015 ... See also: Up-to-date delegate counts each Republican candidate. GOP ... South Carolina Primary (50 delegates): February 20, 2016 — Delegates ... `https://time.com/4059030/republican-primary-calendar-2016-nomination-convention/`

Election Results - Virginia Department of Elections - August 29, 2023 Special Election · June 20, 2023 Democratic Primary Election · June 20, 2023 Republican Primary Election · April 11, 2023 Frederick

County Special ... https://www.elections.virginia.gov/resultsreports/election-results/

The Real Magic Number That Donald Trump Needs to Hit Is Likely ... - Experts weigh in on the delegate count needed for the GOP nomination. ... April 20, 2016, 8:26 AM. 4:02. What's Next in the 2016 Presidential Race. https://abcnews.go.com/Politics/real-magic-number-donald-trump-hit-lower-1237/story?id=38539256

Upcoming Elections | Elections - Through October 16: Register at the Fairfax County Office of Elections; October 16 - November 4: Voters may register at an early voting location and vote a ... https://www.fairfaxcounty.gov/elections/upcoming

2016 Delegate Count Tracker - Jul 24, 2016 ... Democratic Delegates. Candidate view Calendar view. Republican Democratic · Needed to win nomination: 2,383 · Hillary Clinton: 2,814 (includes 609 ... https://www.bloomberg.com/politics/graphics/2016-delegate-tracker/

Who's On Track For The Nomination? | FiveThirtyEight - Tracking a candidate's progress requires more than straight delegate counts. We've estimated how many delegates each candidate would need in each primary ... http://projects.fivethirtyeight.com/election-2016/delegate-targets/

Presidential election in Indiana, 2016 - Ballotpedia - 2008 Democratic winner: Hillary Clinton; Type: Open Primary; Delegate allocation: Proportional; Pledged delegates at stake: 83. Republicans. 2016 Republican ... https://ballotpedia.org/Presidential_election_in_Indiana,_2016

Output: Mostly-true, the statement made by Donald Trump on 4/20/2016 that "Ted Cruz is mathematically out of winning the race" is mostly true. According to the delegate count from the New York Times (nytimes.com), Donald Trump had significantly more delegates than Ted Cruz. However, it's important to note that the race isn't officially over until a candidate reaches the required number of delegates to secure the nomination, which in 2016 was 1,237 delegates (abcnews.go.com). At the time of the statement, Trump had not yet reached this number, so technically Cruz was not mathematically eliminated. However, his path to victory was highly unlikely.

Politifact Ruling: Trump said, "Ted Cruz is mathematically out of winning the race." Cruz would need at the very least 678 more delegates to win the race. It's impossible for him to reach that number in the remaining primaries, given that there are just 674 delegates left to win. However, he could conceivably find more supporters in the states who've already held primaries, given that a few dozen are thus far uncommitted. Trump's statement is largely accurate but needs additional information. We rate it Mostly True. https://www.politifact.com/factchecks/2016/apr/20/donald-trump/trump-largely-accurate-cruz-has-been-mathematicall/

1.2 Example 2 - Trump's limousine driver did burnouts to entertain Daytona 500 spectators during a rain delay

Input: You're a fact-checking expert. Evaluate the truthfulness of this statement: 'Says President Trump's limousine driver did burnouts to entertain Daytona 500 spectators during a rain delay.' made by Viral image on 2/21/2020. Use Google for research. Consider your sources, context, and date while assessing. To answer return 'Final Answer: verdict, reason. You must respond with a valid verdict ('pants-fire','false',

’mostly-false’, ’half-true’, ’mostly-true’, or ’true’) or ’uncertain’, providing reasoning and citing sources by providing the domain of pertinent search results.

Google Search Query: President Trump limousine Daytona 500 burnouts

’Start your engines!’ President Trump revs up Daytona 500 crowd ... - Feb 16, 2020 ... He was the first of any president to take a lap in the armored presidential limo, dubbed "The Beast," FOX News reported. Trump and first lady ... `https://www.fox2detroit.com/news/start-your-engines-president-trump-revs-up-daytona-500-crowd-does-historic-lap-in-presidential-limo`

Denny Hamlin wins 3rd Daytona 500; Ryan Newman hospitalized - Feb 17, 2020 ... President Donald Trump and first lady Melania Trump ride in the presidential limousine as they take a pace lap ahead of the start of the ... `https://www.news4jax.com/sports/2020/02/17/daytona-500-resumes-without-president-pomp-packed-house/`

Ryan Newman hospitalized in horrific wreck as Hamlin wins ... - Feb 17, 2020 ... Denny Hamlin won a crash-filled Daytona 500 on Monday after a ghastly final-lap ... Trump takes lap in armored presidential limo ’the Beast’. `https://www.theguardian.com/sport/2020/feb/17/daytona-500-denny-hamlin-ryan-newman-crash`

NASCAR 2020 Thread | Steeler Nation - Feb 16, 2020 ... President Trump becomes first president named Daytona 500 grand marshal. DAYTONA BEACH, Fla. ... do a burn-out. the-beast-president-limo.jpg ... `https://www.steelernation.com/forums/threads/nascar-2020-thread.34154/`

Daytona 500 race hi-res stock photography and images - Alamy - RM 2B045R1–U.S President Donald Trump and First Lady Melania Trump ride in the presidential limousine as they take a pace lap ahead of the start of the ... `https://www.alamy.com/stock-photo/daytona-500-race.html`

What cars do the presidential candidates drive? Trump, Biden and ... - Mar 9, 2020 ... Trump last month dropped in at the Daytona 500 and did a partial lap riding inside of it before getting out to address NASCAR fans in ... `https://www.autoblog.com/2020/03/09/presidential-candidate-cars-they-drive/`

Daytona 500 hi-res stock photography and images - Alamy - Trump served as the official starter of the the NASCAR Daytona 500 auto race and took a ride in the presidential limousine around the track. `https://www.alamy.com/stock-photo/daytona-500.html`

Cars Of The Presidents - Nov 2, 2020 ... US President Donald Trump and First Lady Melania Trump ride in the ... that may even mean hitting the banked asphalt at the Daytona 500. `https://www.autoblog.com/photos/photos-famous-presidential-cars/`

Cadillac Presidential Limo Cost ’The Beast’ Cadillac Presidential ... - Oct 22, 2020 ... One of The Beast’s more famous appearances was at the Daytona 500 earlier this year, when Trump lapped the Daytona International Speedway in ... `https://gmauthority.com/blog/2020/10/the-beast-cadillac-presidential-limo-costs-53-times-more-than-average-u-s-car/`

Google Search Query: Daytona 500 2020 Trump limousine burnouts

Ryan Newman hospitalized in horrific wreck as Hamlin wins ... - Feb 17, 2020 ... ... 500 at Daytona International Speedway. Photograph: Peter Casey/USA Today Sports. Nascar ... Trump takes lap in armored presidential limo ’the ... `https://www.theguardian.com/sport/2020/feb/17/daytona-500-denny-hamlin-ryan-newman-crash`

Denny Hamlin wins 3rd Daytona 500; Ryan Newman hospitalized - Feb 17, 2020 ... ... burnouts that Hamlin learned the accident was bad. “It’s a weird balance of ... Trump takes Daytona 500 warmup lap in presidential limousine. https://www.news4jax.com/sports/2020/02/17/daytona-500-resumes-without-president-pomp-packed-house/

’Start your engines!’ President Trump revs up Daytona 500 crowd ... - ... !’ President Trump revs up Daytona 500 crowd, does historic lap in ... Deputy gives Dream Cruise driver a spanking after he did burnout on Woodward ... https://www.fox2detroit.com/news/start-your-engines-president-trump-revs-up-daytona-500-crowd-does-historic-lap-in-presidential-limo

NASCAR 2020 Thread | Steeler Nation - Feb 16, 2020 ... Daytona 500 this afternoon. 2:30 on Fox, pre-race is earlier. Word is that President Trump may do a lap in the Presidential limo. https://www.steelernation.com/forums/threads/nascar-2020-thread.34154/

Daytona 500 race hi-res stock photography and images - Alamy - ... Trump and First Lady Melania Trump ride in the presidential limousine as they ... race at Daytona International Speedway February 16, 2020 in Daytona Beach, ... https://www.alamy.com/stock-photo/daytona-500-race.html

What cars do the presidential candidates drive? Trump, Biden and ... - Mar 9, 2020 ... Trump last month dropped in at the Daytona 500 and did a partial lap ... On the back hatch was a new bumper sticker: “Bernie 2020.” Joe Biden. https://www.autoblog.com/2020/03/09/presidential-candidate-cars-they-drive/

Daytona 500 hi-res stock photography and images - Alamy - ... Trump and First Lady Melania Trump ride in the presidential limousine as they ... race at Daytona International Speedway February 16, 2020 in Daytona Beach, ... https://www.alamy.com/stock-photo/daytona-500.html

Cadillac Presidential Limo Cost ’The Beast’ Cadillac Presidential ... - Oct 22, 2020 ... One of The Beast’s more famous appearances was at the Daytona 500 ... Trump and thought you were gonna get a positive response bruh shutup and ... https://gmauthority.com/blog/2020/10/the-beast-cadillac-presidential-limo-costs-53-times-more-than-average-u-s-car/

NASCAR on NBC - One of the coolest sights every year. A flyover by ... - Feb 16, 2020 · . Timeline photos. One of the coolest sights every ... the presidential limo would do a burnout! 4 yrs. 11. Keith Edge. Trump has rally in ... https://www.facebook.com/NASCARonNBC/photos/one-of-the-coolest-sights-every-yeara-flyover-by-the-air-force-thunderbirds-at-t/1835566633246093/

Final Output: False, there is no evidence from reliable sources to suggest that President Trump’s limousine driver performed burnouts at the Daytona 500. The sources only confirm that Trump took a lap in his limousine.

PolitiFact Verdict: Sorry to disappoint, but no. A Facebook post shares an altered image of such a scene, along with a caption that says, "the presidential limo driver entertains the crowd during the rain delays at the Daytona 500 with smokey burnouts.” [...] The image then got passed around as authentic. If such a thing happened in the First Car, we would all know about it. We rate this altered photo an extra-smoky Pants on Fire! https://www.politifact.com/factchecks/2020/feb/21/viral-image/no-presidential-limo-wasnt-doing-burnouts-daytona-/

1.3 Example 3 - The Obama administration gave Iran $400 million in ransom payment cash.

Input: You're a fact-checking expert. Evaluate the truthfulness of this statement: The Obama administration gave Iran $400 million in ransom payment cash. made by Donald Trump on 8/24/2016. Use Google for research. Consider your sources, context, and date while assessing. To answer return 'Final Answer: verdict, reason. You must respond with a valid verdict ('pants-fire','false', 'mostly-false', 'half-true', 'mostly-true', or 'true') or 'uncertain', providing reasoning and citing sources by providing the domain of pertinent search results.

Google Search Query: Obama administration $400 million payment to Iran

Investigating the Obama administration's $400 million payment to Iran - Aug 4, 2016 ... The Wall Street Journal revealed this week that in January, the Obama administration secretly airlifted $400 million in cash to Iran. `https://www.pbs.org/newshour/show/investigating-obama-administrations-400-million-payment-iran`

US sent plane with $400 million in cash to Iran | CNN Politics - Aug 4, 2016 ... The Obama administration secretly arranged a plane delivery of $400 million in cash on the same day Iran released four American prisoners ... `https://www.cnn.com/2016/08/03/politics/us-sends-plane-iran-400-million-cash/index.html`

$400 Million: Why the U.S. Owed Iran in the First Place | Time - Aug 5, 2016 ... Answer: The actual hostage deal that in fact accounts for the cash payment, which President Obama said on Thursday was not a ransom. The ... `https://time.com/4441046/400-million-iran-hostage-history/`

Fact-checking Trump tweets on Obama's payments to Iran | CNN ... - Jun 21, 2019 ... The entire $1.7 billion, given in two separate payments of $400 million and $1.3 billion, was provided in cash. The timing of the first payment ... `https://www.cnn.com/2019/06/21/politics/trump-twitter-iran-fact-check/index.html`

U.S. Concedes $400 Million Payment to Iran Was Delayed as ... - Aug 18, 2016 ... For months the Obama administration had maintained that the payment was part of a settlement over an old dispute and did not amount to a "ransom ... `https://www.nytimes.com/2016/08/19/world/middleeast/iran-us-cash-payment-prisoners.html`

Chairman Chaffetz seeks answers for $400 million sent to iran ... - Aug 3, 2016 ... ... letter to Secretary of State John Kerry asking for information surrounding the Obama administration's $400 million cash payment to Iran. `https://oversight.house.gov/release/chairman-chaffetz-seeks-answers-400-million-sent-iran/`

$400 Million to Iran Was Not a Ransom, Obama Says - The New ... - Aug 4, 2016 ... The president called the uproar over the payment, which was made public at the time as part of a landmark nuclear pact, "the manufacturing ... `https://www.nytimes.com/2016/08/05/world/middleeast/400-million-to-iran-was-not-a-ransom-obama-says.html`

U.S. Transferred $1.3 Billion More in Cash to Iran After Initial Payment - Sep 6, 2016 ... The Obama administration followed up a planeload of $400 million in cash sent to Iran in January with two more such shipments in the next 19 ... `http://www.wsj.com/articles/u-s-sent-two-more-planeloads-of-cash-to-iran-after-initial-payment-1473208256`

Obama administration admits $400M payment to Iran tied to release ... - WASHINGTON - The Obama administration said Thursday that a $400 million cash payment to Iran seven months ago was contingent on the release of a group of ... `https://www.jacksonville.com/story/news/nation-world/2016/08/19/obama-administration-admits-400m-payment-iran-tied-release-prisoners/15721348007/`

The United States, Iran, and $1.7 billion: Sorting out the details ... - Oct 3, 2016 ... As an Obama administration official explained earlier this year, ... of the $400 million principal and payment of $1.3 billion in interest. `https://www.brookings.edu/articles/the-united-states-iran-and-1-7-billion-sorting-out-the-details/`

Google Search Query: Purpose of Obama administration's $400 million payment to Iran

Investigating the Obama administration's $400 million payment to Iran - Aug 4, 2016 ... The Wall Street Journal revealed this week that in January, the Obama administration secretly airlifted $400 million in cash to Iran. `https://www.pbs.org/newshour/show/investigating-obama-administrations-400-million-payment-iran`

US sent plane with $400 million in cash to Iran | CNN Politics - Aug 4, 2016 ... He described the payment as a "conscious strategic decision that was made on the part of the Obama administration as we were implementing the ... `https://www.cnn.com/2016/08/03/politics/us-sends-plane-iran-400-million-cash/index.html`

Chairman Chaffetz seeks answers for $400 million sent to iran ... - Aug 3, 2016 ... ... letter to Secretary of State John Kerry asking for information surrounding the Obama administration's $400 million cash payment to Iran. `https://oversight.house.gov/release/chairman-chaffetz-seeks-answers-400-million-sent-iran/`

U.S. Concedes $400 Million Payment to Iran Was Delayed as ... - Aug 18, 2016 ... For months the Obama administration had maintained that the payment was part of a settlement over an old dispute and did not amount to a "ransom ... `https://www.nytimes.com/2016/08/19/world/middleeast/iran-us-cash-payment-prisoners.html`

$400 Million: Why the U.S. Owed Iran in the First Place | Time - Aug 5, 2016 ... Answer: The actual hostage deal that in fact accounts for the cash payment, which President Obama said on Thursday was not a ransom. The ... `https://time.com/4441046/400-million-iran-hostage-history/`

Senator Scott Joins Rubio to Stop Obama Ransom Payments | U.S. ... - Sep 6, 2016 ... ... In response to the Obama Administration's ransom payments to Iran, ... about a $400 million ransom payment to the Iranian government. `https://www.scott.senate.gov/media-center/press-releases/senator-scott-joins-rubio-to-stop-obama-ransom-payments`

Tillis, Colleagues Demand Answers After Biden Administration ... - Aug 18, 2023 ... "When the Obama administration released $400 million in liquidated assets to Iran in 2016, we warned that this dangerous precedent would put ... `https://www.tillis.senate.gov/2023/8/tillis-colleagues-demand-answers-after-biden-administration-releases-6-billion-to-iran-in-secret-negotiation`

Sen. Moran Statement on Iran Ransom Payment - News Releases ... - Aug 19, 2016 ... ... regarding the Obama Administration's ransom payment to Iran in ... has been clear for many of us – the $400 million in

cash paid to Iran ... https://www.moran.senate.gov/public/index.cfm/news-releases?ID=C3BBED8C-42D3-46A9-BFCF-340E5C852B37

Wagner Grills Administration Officials on Iran Ransom ... - Sep 8, 2016 ... ... more clear that the Obama Administration lied to the American people and sent a cargo plane loaded with $400 million in cash to pay Iran ... https://wagner.house.gov/media-center/press-releases/wagner-grills-administration-officials-iran-ransom

"Something Tangible" - Dr. Boozman's Check-up - Senator John ... - Aug 5, 2016 ... The Obama Administration has some questions to answer about the $400 million cash payment to Iran on the same day it released four American ... https://www.boozman.senate.gov/public/index.cfm/2016/8/something-tangible

Final Output: 'half-true', The Obama administration did send $400 million to Iran, but whether this payment was a "ransom" is disputed and seems to depend on one's interpretation of the term.

Politifact Verdict: Trump said the Obama administration gave Iran "$400 million in ransom payment cash." On the same day in January, the United States paid Iran $400 million dollars, and Iran released several American prisoners. While there appears to be a quid pro quo — the United States would not give Iran the cash until the prisoners were on their way home — this does not equate to ransom, experts told us. [....] Trump's statement contains some element of truth but ignores critical facts that would give a different impression. We rate it Mostly False. https://www.politifact.com/factchecks/2016/aug/24/donald-trump/donald-trump-calls-400-million-payment-iran-ransom/

1.4 Example 4 - Miller Brewing is not a U.S. company any more

Input: You're a fact-checking expert. Evaluate the truthfulness of this statement: '"Miller Brewing is not a U.S. company any more. Neither is Anheuser-Busch."' made by Paul Ryan on 9/8/2014. Use Google for research. Consider your sources, context, and date while assessing. To answer return 'Final Answer: verdict, reason. You must respond with a valid verdict ('pants-fire','false', 'mostly-false', 'half-true', 'mostly-true', or 'true') or 'uncertain', providing reasoning and citing sources by providing the domain of pertinent search results.

Google Search Query: Miller Brewing current ownership and headquarters

Miller Brewing Company - Wikipedia - The Miller Brewing Company is an American brewery and beer company in Milwaukee, Wisconsin. It was founded in 1855 by Frederick Miller. https://en.wikipedia.org/wiki/Miller_Brewing_Company

Molson Coors: Home - The official website of Molson Coors Beverage Company, which for more than two centuries has been brewing beverages that unite people for all of life's ... https://www.molsoncoors.com/

MillerCoors - Wikipedia - MillerCoors was a beer brewing company in the United States. MillerCoors was formed in 2008 as a joint venture between SABMiller and Molson Coors to combine ... https://en.wikipedia.org/wiki/MillerCoors

Office of Public Affairs | Justice Department Requires Anheuser ... - Jul 20, 2016 ... "The two largest U.S. brewers – ABI and MillerCoors – will now remain ... MillerCoors owns and operates 12 breweries in the United States. https://www.justice.gov/opa/pr/justice-department-requires-anheuser-busch-inbev-divest-stake-millercoors-and-alter-beer

Where Is Miller High Life Brewed? (Explained) - BeverageBeaver - Below, we'll tell you more about where the company was first founded, the different breweries and who currently owns Miller High Life. Where is this beer ... `https://beveragebeaver.com/where-is-miller-high-life-brewed-explained/`

Who owns Miller Lite? | The US Sun - May 15, 2023 ... Although the beer brand was launched by Rheingold Brewery, the beverage has seen a shift in owner over the years. Advertisement. Miller Lite ... `https://www.the-sun.com/money/8126578/who-owns-miller-lite/`

Miller Brewing Co. | Milwaukee, WI | Beers | BeerAdvocate - In 2016 SABMiller sold its stake in MillerCoors to Molson Coors, which gained full ownership of the Miller brand portfolio outside the US and Puerto Rico, and ... `https://www.beeradvocate.com/beer/profile/105/`

Miller High Life shipment destroyed over 'Champagne of Beers ... - Apr 22, 2023 ... Molson Coors Beverage Co., which owns the Miller High Life brand, does not currently export it to the EU, and Belgian customs declined to ... `https://www.npr.org/2023/04/22/1171460421/miller-beer-champagne-cans-destroyed`

Miller Lite: Home of the Original Lite Beer - The times change. But some things never do. The best times are spent together with our best friends drinking a few beers. That's why we started brewing Miller ... `https://www.millerlite.com/`

Final Output: False. Miller Brewing Company and Anheuser-Busch are still U.S. companies, although their ownership has changed. (sources: Wikipedia, U.S. Department of Justice, BeerAdvocate, NPR)

PolitiFact Verdict: Ryan cited two well-known beer makers – including one founded in Milwaukee – when asked about inversions. He said the parent companies of Miller and Anheuser-Busch are located overseas. They were part of a long-standing and rapid world-wide consolidation of the major breweries. We rate his statement True. `https://www.politifact.com/factchecks/2014/sep/08/paul-ryan/paul-ryan-says-miller-brewing-and-anheuser-busch-a/`
